# Retraction: The Impact of Sleep Deprivation on Brain Fog, Cognitive Decline, and Cardiovascular Risk in Young Adults

**DOI:** 10.7759/cureus.r224

**Published:** 2026-03-30

**Authors:** Aimen Younas, Sarath Vayolipoyil, Shaheera Raghib, Sher Bano, Abali Wandala, Azmir Ali Khan, Areej Amin, Aima Asim Khan, Syed Muhammad Ali, Javed Iqbal, Muhammad Umar, Jannat Ul Ferdous, Syeda Maryam Zehra Zaidi

**Affiliations:** 1 Internal Medicine, Ayub Medical College, Abbottabad, PAK; 2 General Medicine, Scarborough General Hospital, York and Scarborough Teaching Hospitals NHS Foundation Trust, Scarborough, GBR; 3 General Practice, Baqai Medical University, Karachi, PAK; 4 Clinical Psychology, Brain Wave Research Center, Islamabad, PAK; 5 Clinical Research, DHR Health Institute for Research & Development, Edinburg, USA; 6 Medicine, Universidad Adventista del Plata, Libertador San Martín, ARG; 7 Medicine, King Edward Medical University, Lahore, PAK; 8 Medicine, Rawal Institute of Health Sciences, Islamabad, PAK; 9 Medicine, Fazaia Medical College, Islamabad, PAK; 10 Surgery, Weill Cornell Medicine - Qatar, Doha, QAT; 11 Acute Care Surgery, Hamad General Hospital, Doha, QAT; 12 Nursing, Hamad General Hospital, Doha, QAT; 13 Medicine, Khairpur Medical College, Khairpur, PAK; 14 Respiratory Medicine, University of Chester, Chester, GBR; 15 Medicine, Jinnah Medical & Dental College, Karachi, PAK

This article has been retracted by the Editor-in-Chief due to a copyright violation by the authors concerning the unauthorized use of the Mini-Mental State Examination (MMSE). As a result, this article has been retracted and the article contents have been removed.

